# Human ESC-derived vascular cells promote vascular regeneration in a HIF-1α dependent manner

**DOI:** 10.1093/procel/pwad027

**Published:** 2023-05-09

**Authors:** Jinghui Lei, Xiaoyu Jiang, Daoyuan Huang, Ying Jing, Shanshan Yang, Lingling Geng, Yupeng Yan, Fangshuo Zheng, Fang Cheng, Weiqi Zhang, Juan Carlos Izpisua Belmonte, Guang-Hui Liu, Si Wang, Jing Qu

**Affiliations:** Advanced Innovation Center for Human Brain Protection, National Clinical Research Center for Geriatric Disorders, Xuanwu Hospital Capital Medical University, Beijing 100053, China; Aging Translational Medicine Center, International Center for Aging and Cancer, Beijing Municipal Geriatric Medical Research Center, Xuanwu Hospital, Capital Medical University, Beijing 100053, China; State Key Laboratory of Membrane Biology, Institute of Zoology, Chinese Academy of Sciences, Beijing 100101, China; University of Chinese Academy of Sciences, Beijing 100049, China; Advanced Innovation Center for Human Brain Protection, National Clinical Research Center for Geriatric Disorders, Xuanwu Hospital Capital Medical University, Beijing 100053, China; Aging Translational Medicine Center, International Center for Aging and Cancer, Beijing Municipal Geriatric Medical Research Center, Xuanwu Hospital, Capital Medical University, Beijing 100053, China; Advanced Innovation Center for Human Brain Protection, National Clinical Research Center for Geriatric Disorders, Xuanwu Hospital Capital Medical University, Beijing 100053, China; Aging Translational Medicine Center, International Center for Aging and Cancer, Beijing Municipal Geriatric Medical Research Center, Xuanwu Hospital, Capital Medical University, Beijing 100053, China; Advanced Innovation Center for Human Brain Protection, National Clinical Research Center for Geriatric Disorders, Xuanwu Hospital Capital Medical University, Beijing 100053, China; Aging Translational Medicine Center, International Center for Aging and Cancer, Beijing Municipal Geriatric Medical Research Center, Xuanwu Hospital, Capital Medical University, Beijing 100053, China; Advanced Innovation Center for Human Brain Protection, National Clinical Research Center for Geriatric Disorders, Xuanwu Hospital Capital Medical University, Beijing 100053, China; Aging Translational Medicine Center, International Center for Aging and Cancer, Beijing Municipal Geriatric Medical Research Center, Xuanwu Hospital, Capital Medical University, Beijing 100053, China; State Key Laboratory of Membrane Biology, Institute of Zoology, Chinese Academy of Sciences, Beijing 100101, China; Institute for Stem Cell and Regeneration, CAS, Beijing 100101, China; The Fifth People’s Hospital of Chongqing, Chongqing 400062, China; University of Chinese Academy of Sciences, Beijing 100049, China; National Laboratory of Biomacromolecules, CAS Center for Excellence in Biomacromolecules, Institute of Biophysics, Beijing 100101, China; University of Chinese Academy of Sciences, Beijing 100049, China; Institute for Stem Cell and Regeneration, CAS, Beijing 100101, China; CAS Key Laboratory of Genomic and Precision Medicine, Beijing Institute of Genomics, Chinese Academy of Sciences, Beijing 100101, China; China National Center for Bioinformation, Beijing 100101, China; Sino-Danish College, University of Chinese Academy of Sciences, Beijing 101408, China; Sino-Danish Center for Education and Research, Beijing 101408, China; Aging Biomarker Consortium, China; Altos Labs, San Diego, CA 92121, United States; Advanced Innovation Center for Human Brain Protection, National Clinical Research Center for Geriatric Disorders, Xuanwu Hospital Capital Medical University, Beijing 100053, China; State Key Laboratory of Membrane Biology, Institute of Zoology, Chinese Academy of Sciences, Beijing 100101, China; University of Chinese Academy of Sciences, Beijing 100049, China; Institute for Stem Cell and Regeneration, CAS, Beijing 100101, China; Beijing Institute for Stem Cell and Regenerative Medicine, Beijing 100101, China; Aging Biomarker Consortium, China; Advanced Innovation Center for Human Brain Protection, National Clinical Research Center for Geriatric Disorders, Xuanwu Hospital Capital Medical University, Beijing 100053, China; Aging Translational Medicine Center, International Center for Aging and Cancer, Beijing Municipal Geriatric Medical Research Center, Xuanwu Hospital, Capital Medical University, Beijing 100053, China; The Fifth People’s Hospital of Chongqing, Chongqing 400062, China; Aging Biomarker Consortium, China; State Key Laboratory of Stem Cell and Reproductive Biology, Institute of Zoology, Chinese Academy of Sciences, Beijing 100101, China; University of Chinese Academy of Sciences, Beijing 100049, China; Institute for Stem Cell and Regeneration, CAS, Beijing 100101, China; Beijing Institute for Stem Cell and Regenerative Medicine, Beijing 100101, China; Aging Biomarker Consortium, China

**Keywords:** HIF-1, human ESC, vascular cell, regeneration

## Abstract

Hypoxia-inducible factor (HIF-1α), a core transcription factor responding to changes in cellular oxygen levels, is closely associated with a wide range of physiological and pathological conditions. However, its differential impacts on vascular cell types and molecular programs modulating human vascular homeostasis and regeneration remain largely elusive. Here, we applied CRISPR/Cas9-mediated gene editing of human embryonic stem cells and directed differentiation to generate HIF-1α-deficient human vascular cells including vascular endothelial cells, vascular smooth muscle cells, and mesenchymal stem cells (MSCs), as a platform for discovering cell type-specific hypoxia-induced response mechanisms. Through comparative molecular profiling across cell types under normoxic and hypoxic conditions, we provide insight into the indispensable role of HIF-1α in the promotion of ischemic vascular regeneration. We found human MSCs to be the vascular cell type most susceptible to HIF-1α deficiency, and that transcriptional inactivation of *ANKZF1*, an effector of HIF-1α, impaired pro-angiogenic processes. Altogether, our findings deepen the understanding of HIF-1α in human angiogenesis and support further explorations of novel therapeutic strategies of vascular regeneration against ischemic damage.

## Introduction

Ischemic conditions are characterized by reduced blood flow and consequent insufficient oxygen and nutrient supply, causing severe tissue injury (Lei et al., 2021; Van Nguyen et al., 2021). If not resolved quickly, low levels of intracellular ATP and acidic pH levels trigger exacerbated calcium influx in plasma and mitochondria, ultimately causing cell death (Kalogeris et al., 2012). To ameliorate the harsh microenvironment of low oxygen supply and nutrient deprivation caused by ischemia, promotion of angiogenesis to restore the blood flow is considered a promising therapeutic approach (Wahlberg, 2003; Vrselja et al., 2019; Wang and Qin, 2023). However, current clinical therapies such as thrombolytic or vasodilator drugs and surgery fall significantly short of promoting angiogenesis and vascular remodeling efficiently (Bian et al., 2019), and therefore, there is a vast need to investigate mechanistic underpinnings of vascular regeneration for directing development of effective treatments.

Blood vessel mainly consists of three layers including the innermost tunica intima, the middle tunica media, and the outermost tunica adventitia, which are primarily composed of three cell types: vascular endothelial cells (VECs), vascular smooth muscle cells (VSMCs), and mesenchymal stem cells (MSCs) (Wang et al., 2018; Ling et al., 2019; Yan et al., 2019). In particular, MSCs located in the adventitia layer and commonly referred to as vascular wall-resident MSCs are critical for local capacity of neovascularization in disease processes (Ergun et al., 2011; Worsdorfer et al., 2017). Vascular cell activation and endogenous angiogenesis are essential to recover the oxygen supply and boost the repair of the ischemia-induced injured tissues. Several angiogenic growth factors such as VEGF, PDGF, and FGF2 are known to be upregulated upon ischemic insult and act on the corresponding receptors in vascular beds, consequently inducing sprouting and capillary growth toward the ischemic tissue (Dor and Keshet, 1997; Vimalraj, 2022). However, the molecular mechanisms intrinsic to the human vascular cell types underlying ischemic vascular remodeling remain largely unexplored.

Hypoxia-inducible factor (HIF-1α) is a central transcription factor that detects cellular oxygen levels and rapidly responds pathophysiological ischemia. Different from its dimerized partner, constitutively expressed β-subunit (HIF-1β), HIF-1α is sensitive to changes in oxygen levels. Under normoxia, HIF-1α proteins are rapidly hydroxylated by prolyl hydroxylase domain enzymes and degraded. However, hypoxia inhibits the hydroxylation of HIF-1α, preventing its degradation and leading to its accumulation and translocation into the nucleus (Maxwell et al., 1999; Ivan et al., 2001; Jaakkola et al., 2001). In the nucleus, HIF-1α promotes angiogenesis by transcriptionally activating the expression of canonical pro-angiogenic factors, including *VEGF*, *PLGF*, *PDGFB*, and *ANGPT1*, and pro-angiogenic chemokines and receptors, such as *SDF-1*, *S1P*, *CXCR4*, and *S1PR* (Zimna and Kurpisz, 2015; Cai et al., 2022; Feng et al., 2022; Vimalraj, 2022). However, clinical trials have demonstrated that supplementation of these angiogenic factors is usually insufficient to relieve ischemic diseases (Annex and Cooke, 2021). Most importantly, how HIF-1α regulates the physiological functions of different human vascular cells and what the downstream genes of HIF-1α intrinsic to vascular cells are, remain enigmatic. Consequently, gaining insights into such mechanisms are of great importance for developing new therapeutic approaches for ischemic damage and associated diseases.

In this study, we used CRISPR/Cas9-mediated gene editing to generate HIF-1α-deficient human embryonic stem cells (hESCs) and subsequently differentiated these into VECs, VSMCs, and MSCs, the three major vascular cell types. Our data uncovered that human ESC-derived vascular cells promote ischemic vascular regeneration and rescue ischemic damage in a HIF-1α dependent manner. Strikingly, MSCs exhibited the highest susceptibility to HIF-1α deficiency. Through molecular profiling across the vascular cell types, we identified *ANKZF1* as a major effector gene downstream of HIF-1α in mediating angiogenesis in MSCs. Overall, this study identifies novel therapeutic targets for development of approaches to promote vascular regeneration and counteract ischemic diseases.

## Results

### Generation and characterization of HIF-1α-deficient hESCs

**Figure 1.**
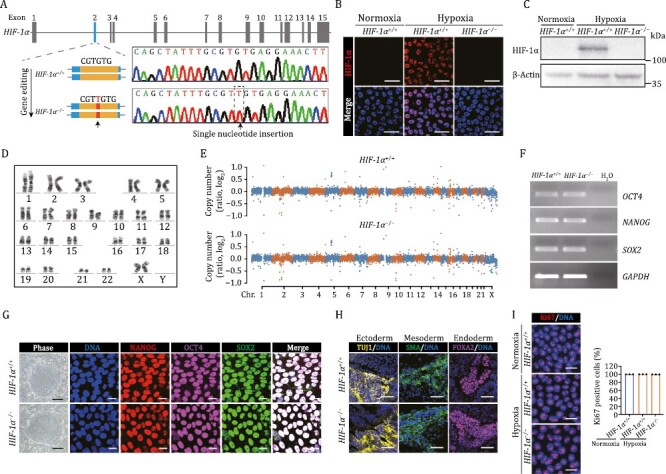
**Generation and characterization of HIF-1α-deficient hESCs.** (A) Schematic illustration of *HIF-1α* gene editing using CRISPR/Cas9-mediated nonhomologous end joining (NHEJ) in hESCs, and a DNA sequence chromatogram showing the single nucleotide insertion within exon 2. (B and C) The expression of HIF-1α was detected in *HIF-1α*^+/+^ and *HIF-1α*^−/−^ hESCs via immunostaining (B) and Western bloting (C). Scale bars, 25 μm. (D) Karyotyping analysis of *HIF-1α*^−/−^ hESCs. (E) The CNV analysis in *HIF-1α*^+/+^ and *HIF-1α*^−/−^ hESCs. (F) The mRNA levels of pluripotency markers *NANOG, OCT4, SOX2* were detected by RT-PCR in *HIF-1α*^+/+^ and *HIF-1α*^−/−^ hESCs. *GAPDH* was used as a loading control. (G) Representative colony morphology and immunostaining of pluripotency markers in *HIF-1α*^+/+^ and *HIF-1α*^−/−^ hESCs. Scale bars, 250 μm for phase and 20 μm for immunostaining. (H) Immunofluorescence staining of TUJ1 (ectoderm), SMA (mesoderm), and FOXA2 (endoderm) in teratomas derived from *HIF-1α*^+/+^ and *HIF-1α*^−/−^ hESCs. Scale bars, 50 μm. (I) Immunofluorescence analysis of Ki67 expression in *HIF-1α*^+/+^ and *HIF-1α*^−/−^ hESCs under normoxic and hypoxic conditions. Data are presented as the mean ± SEM, *n* = 3 independent cell culture wells. Scale bars, 25 μm.

To dissect the role of *HIF-1α* in human vascular cells, we first generated HIF-1α-deficient hESCs (*HIF-1α*^−/−^ hESCs) by targeting exon 2 of the *HIF-1α* gene via CRISPR/Cas9-mediated genome editing (Fig. 1A). Genomic polymerase chain reaction (PCR) and DNA sequencing verified a successful targeting of the *HIF-1α* locus with a single nucleotide insertion (Fig. 1A). In order to induce HIF-1α expression, we cultured human vascular cells in 3% O_2_ to mimic hypoxic conditions *in vitro* (Capitano et al., 2021). Using this protocol, we validated by immunofluorescence staining and Western blot assay that the HIF-1α protein in edited hESCs was absent (Fig. 1B and 1C). The genomic integrity was well maintained in *HIF-1α*^−/−^ hESCs, as confirmed by karyotype and genome-wide copy number variation (CNV) analyses (Fig. 1D and 1E). *HIF-1α*^−/−^ hESCs also maintained normal morphology and expressed pluripotency markers OCT4, SOX2, and NANOG at the same levels as *HIF-1α*^+/+^ hESCs (Fig. 1F and 1G). In teratoma formation assay, we demonstrated that *HIF-1α*^−/−^ hESCs maintained multi-differentiation potential *in vivo* (Fig. 1H), and through Ki67 staining, that HIF-1α deficiency did not compromise hESC proliferation ability (Fig. 1I). Altogether, these data suggest that *HIF-1α*^−/−^ hESCs manifest the typical features of hESCs.

### HIF-1α deficiency impedes the angiogenesis in human vascular cells

**Figure 2.**
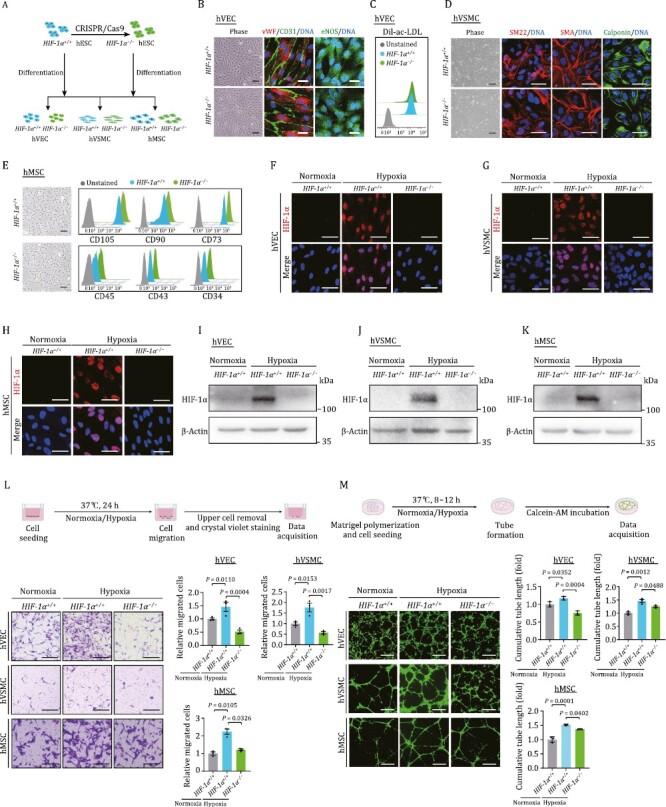
**HIF-1α deficiency impedes human vascular cell functions *in vitro*.** (A) Schematic diagram of the generation of *HIF-1α*^−/−^ hESCs, and the subsequently generation of hVECs, hVSMCs, and hMSCs. (B) Phase images and immunofluorescence staining of hVEC specific markers (CD31, vWF, and eNOS) in *HIF-1α*^+/+^ and *HIF-1α*^−/−^ hVECs. DNA was labeled by Hoechst 33342. Scale bars, 25 μm. (C) Fluorescence-activated cell sorting (FACS) analysis of Dil-ac-LDL uptake ability of *HIF-1α*^+/+^ and *HIF-1α*^−/−^ hVECs. (D) Phase images and immunofluorescence staining of VSMC specific markers (SM22, SMA, and Calponin) in *HIF-1α*^+/+^ and *HIF-1α*^−/−^ hVSMCs. DNA was labeled by Hoechst 33342. Scale bars, 25 μm. (E) Phase images and FACS analysis of hMSC specific markers (CD105, CD90, and CD73) and hMSC irrelevant markers (CD45, CD43, and CD34) in *HIF-1α*^+/+^ and *HIF-1α*^−/−^ hMSCs. Scale bars, 25 μm. (F–H) Representative immunofluorescence images of the HIF-1α expression in hESC-derived hVEC (F), hVSMC (G), and hMSC (H) under normoxic or hypoxic condition. Scale bars, 25 μm. (I–K) Western blot detection of HIF-1α expression in hESC-derived hVEC (I), hVSMC (J), and hMSC (K) under normoxic or hypoxic condition. (L) Examination of HIF-1α deficiency on migration ability in hESC-derived human vascular cells. Upper, experimental schematic of transwell assay. Lower, representative images and quantitative data of relative migration ability in hESC-derived hEC, hVSMC, and hMSC. Scale bars, 200 μm. Data are shown as the mean ± SEM. *n* = 3 independent cell culture wells. One-way ANOVA followed by Tukey’s test. (M) Examination of HIF-1α deficiency on tube formation capacity in hESC-derived human vascular cells. Upper, experimental schematic of tube formation assay. Lower, representative immunofluorescence images and quantitative data of relative tube formation capacity in hESC-derived hVEC, hVSMC, and hMSC. Scale bars, 500 μm. Data are shown as the mean ± SEM. *n* = 3 independent cell culture wells. One-way ANOVA followed by Tukey’s test.

To investigate how HIF-1α deficiency affects human vascular cell function, we performed directed differentiation of *HIF-1α*^−/−^ hESCs to generate HIF-1α-depleted human vascular cells, including human VECs (hVECs), human VSMCs (hVSMCs), and human MSCs (hMSCs) (Fig. 2A). Then, through the use of immunofluorescence or flow cytometric analysis, we validated the identities of the differentiated vascular cells. We purified both *HIF-1α*^+/+^ and *HIF-1α*^−/−^ hVECs via fluorescent-activated cell sorting (FACS) of CD201- and CD144-positive cells (Wang et al., 2022c; Zhao et al., 2023) (see Methods), and found that both cell types expressed comparable levels of canonical VEC markers, such as vWF, CD31, and eNOS (Fig. 2B). Additionally, the functional characterization of VECs, uptake of acetylated low-density lipoprotein (ac-LDL) (Yan et al., 2019), examined using Dil labeled ac-LDL, was similar in *HIF-1α*^+/+^ and *HIF-1α*^−/−^ hVECs (Fig. 2C). Similarly, immunofluorescence staining demonstrated that *HIF-1α*^−/−^ hVSMCs expressed the classic markers including SM22, Calponin, and SMA as did HIF-1α^+/+^ hVSMCs (Wang et al., 2022c) (Fig. 2D). In addition, both *HIF-1α*^+/+^ and *HIF-1α*^−/−^ hMSCs expressed classical MSC surface markers CD105, CD73, and CD90, but were negative for MSC-irrelevant markers CD34, CD43, and CD45 (Cheng et al., 2019; Liang et al., 2021; Wang et al., 2022b) (Fig. 2E). Next, HIF-1α protein expression induced by hypoxia in each cell type was analyzed both by immunofluorescence staining and Western bloting and found to be absent in *HIF-1α*^−/−^ cells (Fig. 2F–K). Collectively, these findings suggested that HIF-1α deficiency does not influence the differentiation capabilities towards human vascular cells.

We subsequently sought to explore in which way HIF-1α contributes to the angiogenic potential of human vascular cells. It is well known that cell migration and *in vitro* formation of capillary-like tubes are crucial for angiogenesis (Zhang et al., 2020b; Ghaffari-Makhmalbaf et al., 2021; Wang et al., 2022a). First, we examined cell migration capability and observed enhanced cellular migration in wild-type (WT) hVECs, hVSMCs, and hMSCs in response to hypoxia compared to normoxic conditions (Fig. 2L). As expected, cellular migration upon induction of hypoxia was compromised in all three types of human HIF-1α-ablated vascular cells (Fig. 2L). And, consistent with the notion that hypoxic condition boots angiogenesis (Pugh and Ratcliffe, 2003), we noticed an increment in the cumulated tube length in WT human vascular cells upon exposure to hypoxia (Fig. 2M). However, the tube formation in HIF-1α-deficient human vascular cells was impaired relative to their WT counterparts, as evidenced by the diminished cumulated tube lengths (Fig. 2M). Overall, these findings elucidated that the HIF-1α signaling cascade is indispensable for hypoxia-induced human vascular cell activation and angiogenis.

### Vascular remodeling and repair upon ischemia damage are compromised in HIF-1α-deficient human vascular cells

**Figure 3.**
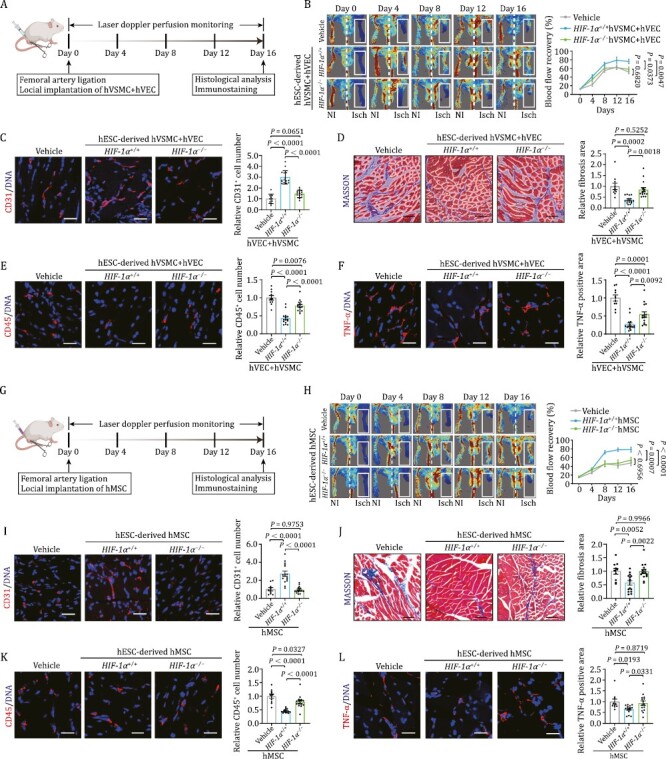
**Human vascular cells repair ischemic damage and suppress inflammatory response in a HIF-1α dependent manner.** (A) Schematic diagram of the experimental procedures for hVEC and hVSMC transplantation. (B) Representative blood flow images and kinetics of hindlimb ischemic mice injected with vehicle, *HIF-1α*^+/+^ and *HIF-1α*^−/−^ cells (hVECs: hVSMCs = 3:1). Laser doppler blood perfusion was measured every 4 days to monitor hindlimb blood flow changes. Data are presented as mean ± SEM. *n*=10 mice for vehicle group, *n* = 15 mice for the other groups. Two-way ANOVA followed by Sidak’s test. (C) Representative images of CD31 immunostaining in ischemic hindlimb sections after implantation of *HIF-1α*^+/+^ or *HIF-1α*^−/−^ cells (hVECs: hVSMCs = 3:1). Scale bars, 25 μm. Quantitative data are shown as the mean ± SEM. *n* = 10 mice for vehicle group, *n* = 15 mice for the other groups. One-way ANOVA followed by Tukey’s test. (D) Representative images of Masson’s trichrome staining in ischemic hindlimb sections after implantation of *HIF-1α*^+/+^ or *HIF-1α*^−/−^ cells (hVECs: hVSMCs = 3:1). Scale bars, 25 μm. Quantitative data are shown as the mean ± SEM. *n*=10 mice for vehicle group, *n* = 15 mice for the other groups. One-way ANOVA followed by Tukey’s test. (E) Representative images of immunostaining of CD45 in ischemic hindlimb sections after implantation of *HIF-1α*^+/+^ or *HIF-1α*^−/−^ cells (hVECs: hVSMCs = 3:1). Scale bars, 25 μm. Quantitative data are shown as the mean ± SEM. *n* = 10 mice for vehicle group, *n* = 15 mice for the other groups. One-way ANOVA followed by Tukey’s test. (F) Representative images of immunostaining of TNF-α in ischemic hindlimb sections after delivery of *HIF-1α*^+/+^ or *HIF-1α*^−/−^ cells (hVECs: hVSMCs = 3:1). Scale bars, 25 μm. Quantitative data are shown as the mean ± SEM. *n* = 10 mice for vehicle group, *n* = 15 mice for the other groups. One-way ANOVA followed by Tukey’s test. (G) Schematic diagram of the experimental procedures for hMSC transplantation. (H) Representative blood flow images and kinetics of hindlimb ischemic mice injected with vehicle, *HIF-1α*^+/+^ hMSCs and *HIF-1α*^−/−^ hMSCs. Laser doppler blood perfusion was measured every 4 days to monitor hindlimb blood flow changes. Data are presented as mean ± SEM. *n* = 10 mice for vehicle group, *n* = 15 mice for the other groups. Two-way ANOVA followed by Sidak’s test. (I) Representative images of immunostaining of CD31 in ischemic hindlimb sections after implantation of *HIF-1α*^+/+^ or *HIF-1α*^−/−^ hMSCs. Scale bars, 25 μm. Quantitative data are shown as the mean ± SEM. *n* = 10 mice for vehicle group; *n* = 15 mice for the other groups. One-way ANOVA followed by Tukey’s test. (J) Representative images of Masson’s trichrome staining in ischemic hindlimb sections after implantation of *HIF-1α*^+/+^ or *HIF-1α*^−/−^ hMSCs. Scale bars, 25 μm. Quantitative data are shown as the mean ± SEM. *n* = 10 for vehicle group; *n* = 15 for the other groups. One-way ANOVA followed by Tukey’s test. (K) Representative images of immunostaining of CD45 in ischemic hindlimb sections after implantation of *HIF-1α*^+/+^ or *HIF-1α*^−/−^ hMSCs. Scale bars, 25 μm. Quantitative data are shown as the mean ± SEM. *n* = 10 mice for vehicle group; *n* = 15 mice for the other groups. One-way ANOVA followed by Tukey’s test. (L) Re presentative images of immunostaining of TNF-α in ischemic hindlimb sections after implantation of *HIF-1α*^+/+^ or *HIF-1α*^−/−^ hMSCs. Scale bars, 25 μm. Quantitative data are shown as the mean ± SEM. *n* = 10 mice for vehicle group; *n* = 15 mice for the other groups. One-way ANOVA followed by Tukey’s test.

Next, to inspect the function of HIF-1α in human vascular cells on neovascularization *in vivo*, we used a well-established murine model of hindlimb ischemia with femoral artery ligation (Yang et al., 2017; Yan et al., 2019). First, we performed the laser doppler perfusion monitoring assay to measure the local microcirculatory blood perfusion after surgery (Fig. 3A). Compared with hindlimbs without femoral artery ligation, block of blood flow was noticed in the surgery group (Fig. 3B). Intriguingly, when we measured the local microcirculatory blood at different time points after cell implantation, we found that implantation of a mixture of *HIF-1α*^+/+^ hVECs and hVSMCs into the ischemic legs led to a more rapid recovery of blood flow compared to those implanted with *HIF-1α*^−/−^ cells (Fig. 3B). These observations suggest that HIF-1α deficiency impairs the angiogenesis-promoting beneficial effect of human vascular cells. In accordance with the aforementioned observations, capillary density, as indicated by CD31-positive cells, was also remarkably increased by implantation of *HIF-1α*^+/+^ cells relative to *HIF-1α*^−/−^ cells at the tissue level (Fig. 3C). Concurrently, we observed that ischemia-induced increase in fibrosis, a hallmark feature of ischemic damage (Stabile et al., 2003; Zhang et al., 2022a), was diminished upon *HIF-1α*^+/+^ cell implantation relative to what we observed in the *HIF-1α*^−/−^ cell delivery group (Fig. 3D). Since it is well known that ischemia induces inflame ischemic lesions (Eltzschig and Carmeliet, 2011), we next examined inflammation levels of ischemic tissues with or without human vascular cell transplantation. Immunofluorescence staining of CD45, a pan-marker for immune cells (Altin and Sloan, 1997; Geng et al., 2022), showed that the infiltration of CD-45 positive immune cells in the hindlimb was markedly alleviated by delivery of a mixture of *HIF-1α*^+/+^ hVECs and hVSMCs to local lesions relative to delivery of *HIF-1α*^−/−^ counterparts (Fig. 3E). More strikingly, TNF-α positive area was also less in the *HIF-1α*^+/+^ cell-implanted group compared to that in *HIF-1α*^−/−^ group (Fig. 3F). Collectively, these data indicated that HIF-1α ablation compromises the pro-angiogenic role of human vascular cell function under ischemic condition.

It is well accepted that activation of MSC-like cells in adventitial wall also play a critical role on vascular protection and regeneration (Vono et al., 2012). Indeed, when implanted into ischemic hindlimb, we found that *HIF-1α*^+/+^ hMSCs but not *HIF-1α*^−/−^ hMSCs induced a superior recovery of blood perfusion in the hindlimb ischemia mouse model (Fig. 3G and 3H). Consistently, capillary density, as assessed by quantification of CD31-positive cells in hindlimb muscles, was only increased after transplantation of *HIF-1α*^+/+^ hMSCs compared to the Vehicle control (Fig. 3I). Moreover, ischemia-induced limb fibrosis was ameliorated by *HIF-1α*^+/+^ hMSC alone (Fig. 3J). In addition, *HIF-1α*^+/+^ hMSCs also attenuated the ischemia-induced inflammation characterized by massive infiltration of CD45-positive immune cells and elevated release of inflammatory cytokine TNF-α, which was not the case upon transplantation of *HIF-1α*^−/−^ hMSCs (Fig. 3K and 3L). Altogether, these data indicated that hMSC transplantation both augments angiogenesis and blunts fibrosis and inflammation after ischemia, while knockout of HIF-1α abrogates the angiogenic and therapeutic potential of hMSCs.

### Transcriptomic analysis reveals HIF-1α-dependent and cell type-specific molecular signatures

**Figure 4.**
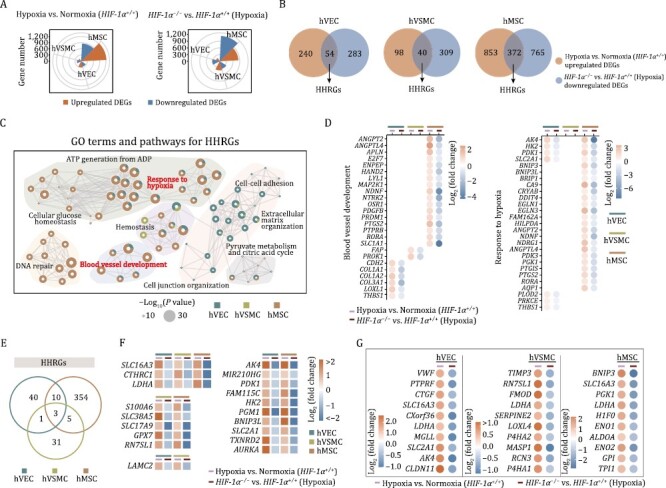
**Transcriptomic analysis reveals the role of HIF-1α in human vascular cells.** (A) Wind rose plots showing DEGs numbers between *HIF-1α*^+/+^ cells (hVECs, hVSMCs, and hMSCs) under normoxic and hypoxic condition (left), or between *HIF-1α*^+/+^ and *HIF-1α*^−/−^ cells (hVECs, hVSMCs, and hMSCs) under hypoxic condition (right). (B) Venn diagrams showing the number of upregulated hypoxia-associated DEGs and the number of downregulated DEGs in human vascular cells with HIF-1α deficiency. The number of indicated overlapping genes was also shown and defined as “hypoxia-induced HIF-1α responsive genes (HHRGs)”. (C) GO term and pathway enrichment analysis of HHRGs across different human vascular cells. (D) Bubble plot showing the relative expression levels of DEGs associated with indicated terms and pathways enriched in panel (C). (E) Venn diagram showing the number of overlapping genes or cell type-specific genes of HHRGs in hVECs, hVSMCs, and hMSCs. (F) Heatmaps showing the relative expression levels of DEGs in two or three cell types. (G) Dot plots showing the relative expression levels of HHRGs in hVECs, hVSMCs, and hMSCs.

HIF-1α exerts biological activities primarily via transcriptional activation of its target genes (Masoud and Li, 2015). To understand such activities in human vascular cell types, we performed whole-genome RNA-seq in *HIF-1α*^+/+^ and *HIF-1α*^−/−^ cells under normoxic and hypoxic conditions. As confirmed by principal component analysis (PCA) and vascular cell type-specific transcriptomic signatures, the replicates within each group were highly reproducible (Fig. S1A–C). Expectedly, hypoxia induced a panel of canonical genes with a functional enrichment of “blood vessel development” (e.g., *VEGFA*, *ANGPT2*, and *THBS1*) and “response to decreased oxygen levels” (e.g., *HIF3A*, *HK2*, and *AK4*) across different vascular cells (Fig. S1D). However, from a global view, hypoxia and the absence of HIF-1α influenced the transcriptomic programs in the human vascular cell types differently (Figs. 4A and S1E). Specifically, we found that hMSC was the most sensitive cell type both to hypoxia and HIF-1α abrogation, as evidenced by the greatest numbers of hypoxia-induced differentially expressed genes (DEGs) in *HIF-1α*^+/+^ cells, as well as DEGs between *HIF-1α*^−/−^ and *HIF-1α*^+/+^ cells under hypoxic condition (Fig. 4A). Specifically, hypoxia induced 2082, 553, and 343 total DEGs in *HIF-1α*^+/+^ hMSCs, hVECs, and hVSMCs, respectively (1225, 294, 138 upregulated and 857, 259, 205 downregulated DEGs in hMSCs, hVECs, and hVSMCs, respectively) (Fig. S1E and Table S1). Similarly, depletion of HIF-1α in the presence of hypoxia resulted in a more pronounced transcriptional perturbation in hMSCs, inducing 1977 total DEGs in hMSCs (840 upregulated and 1137 downregulated) relative to 506 (169 upregulated and 337 downregulated) and 760 (411 upregulated and 349 downregulated) total DEGs in hVECs and hVSMCs, respectively (Fig. S1E and Table S1).

Next, we focused on analyzing overlapping genes between upregulated DEGs by hypoxia exposure in WT cells and downregulated ones in *HIF-1α*^−/−^ vs. *HIF-1α*^+/+^ cells after hypoxia exposure, which we referred to as hypoxia-induced HIF-1α responsive genes (HHRGs) (Fig. 4B). As shown by Venn diagram, hMSCs contained the most HHRGs (54 genes in hVECs, 40 genes in hVSMCs, and 372 genes in hMSCs, respectively) (Fig. 4B), which was concordant with the highest transcriptional fluctuations observed in hMSCs (Fig. 4A). Through Gene Ontology (GO) term and pathway enrichment analysis, we discovered that although these HHRGs were divergent across three cell types (Fig. 4B and 4D), they functionally converged on “blood vessel development” (e.g., *ANGPT2* and *ANGPTL4* in hMSCs*, FAP* and *PROK1* in hVSMCs, *LOXL1* and *THBS1* in hVECs) and “response to hypoxia” (e.g., *AK4*, *HK2*, *PDK1*, and *SLC2A1* in hVECs and hMSCs, *PLOD2* and *PRKCE* in hVECs) (Figs. 4C, 4D and S1F). Notably, three classical HIF-1α target genes (*SLC16A3*, *CTHRC1*, and *LDHA*) were shared across all three cell types (Fig. 4E and 4F). Among these, *SLC16A3* encodes a member of the solute carrier family-16, which catalyzes lactic acid and pyruvate transport across the plasma membranes (Contreras-Baeza et al., 2019); *CTHRC1* encodes a secretory protein, collagen triple helix repeat containing 1, which is involved in the cellular response to arterial injury through facilitation of vascular remodeling (Pyagay et al., 2005). Moreover, amongst the top 10-ranked HHRGs of different vascular cells, some were shared across three cell types and some exhibited cell type specificity (Fig. 4G). For example, *LDHA*, encoding lactate dehydrogenase A that catalyzes the conversion of l-lactate and nicotinamide adenine dinucleotide to pyruvate and hydrogenated nicotinamide adenine dinucleotide, was shared by three cell types, which suggested its central role in anaerobic glycolysis closely relevant to vessel sprouting (De Bock et al., 2013; Valvona et al., 2016; Du et al., 2021). *VWF*, specific to hVECs, encodes a glycoprotein responsible for hemostasis by promoting adhesion of platelets to the sites of vascular injury (Chen and Lopez, 2005). Overall, our data revealed that HIF-1α transcriptionally activated different sets of downstream genes under hypoxia condition, which may convergently mediate pro-angiogenic functions in human vascular cells.

### *ANKZF1* acts as an effector gene downstream of *HIF-1α* in hMSCs

**Figure 5.**
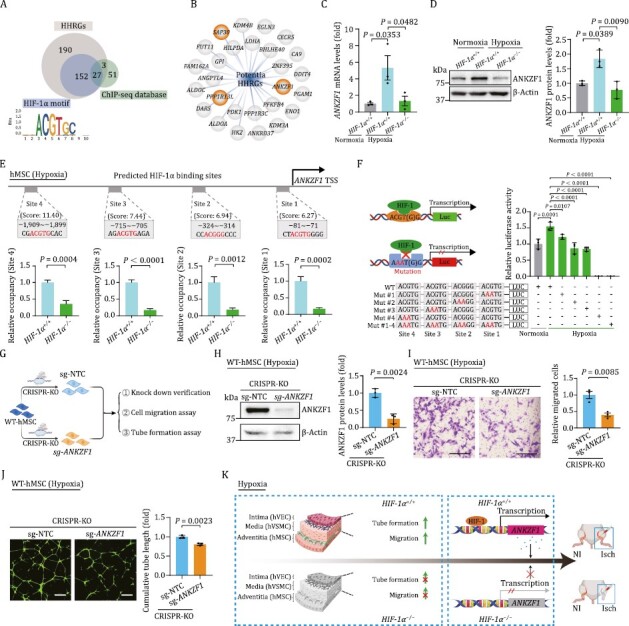
**ANKZF1 acts as a major effector gene downstream of HIF-1α in hMSCs.** (A) Conjoint analysis of HHRGs containing the canonical HIF-1α binding motif and the genes harboring the potential HIF-1α binding sites from ChIP-seq database. (B) 27 overlapped genes in panel (A) were exhibited as potential HHRGs in hMSCs by network plot diagram. Three genes with orange background were referred to as novel HHRGs in hMSCs (C) RT-qPCR verified the changes of *ANKZF1* mRNA level upon HIF-1α depletion in hMSCs. Data are shown as the mean ± SEM. *n* = 3 independent experiments. One-way ANOVA followed by Tukey’s test. (D) Western blot analysis of ANKZF1 upon HIF-1α depletion in hMSCs. Data are shown as the mean ± SEM. *n* = 3 independent experiments. One-way ANOVA followed by Tukey’s test. (E) ChIP-qPCR analysis showing the binding of HIF-1α to *ANKZF1* promoter. Data were presented as mean ± SEM. Two-tailed Student’s *t* test. (F) Diagram (left) and quantitative data (right) of luciferase reporter assay of HIF-1α on the luciferase activity expressed from *ANKZF1* promoter. Data are shown as the mean ± SEM. *n* = 3 independent cell culture wells. One-way ANOVA followed by Tukey’s test. (G) Schematic diagram showing the CRSIPR/Cas9-mediated knockout of *ANKZF1* in hMSCs and subsequent related experiments. (H) Western blot showing the protein level of ANKZF1 in hMSCs transduced with nontargeting (sg-NTC) or *ANKZF1* targeting (sg-*ANKZF1*) sgRNA. Left, representative images of Western blot. Right, statistical analysis of relative protein levels of ANKZF1. β-actin was used as the loading control. Data are presented as the mean ± SEM, *n* = 3 independent experiments. Two-tailed Student’s *t* test. (I) Representative images and quantitative data of cell migration assay in hMSCs transduced with sg-NTC or sg-*ANKZF1* sgRNA. Scale bars, 200 μm. *n* = 3 independent cell culture wells. Data are shown as the mean ± SEM. Two-tailed Student’s *t* test. (J) Representative images and quantitative data of tube formation assay in hMSCs transduced with sg-NTC or sg-*ANKZF1* sgRNA. Scale bars, 500 μm. *n* = 3 independent cell culture wells. Data are shown as the mean ± SEM. Two-tailed Student’s *t* test. (K) A schematic illustration showing the pro-angiogenetic effects of HIF-1α on human vascular cells *in vitro* and *in vivo*, and identified ANKZF1 as a novel HIF-1α target gene in human vascular cells.

Given the highest susceptibility of hMSCs manifested by altered transcriptomic profiling, we next explored the mechanism underpinning HIF-1α-mediated pro-angiogenic capacity in hMSCs. Through a conjoint analysis of HHRGs containing the canonical HIF-1α binding motif and the genes harboring the potential HIF-1α binding sites from ChIP-seq database (Rouillard et al., 2016; Zhang et al., 2020a), we identified 27 genes as potential HHRGs in hMSCs (Fig. 5A and 5B). Among these, 24 genes have been identified as HIF-1α target genes by other studies (Lee et al., 2004; Masoud and Li, 2015), while the other three genes were unreported and therefore referred to as novel HHRGs in hMSCs (Fig. 5B). Consistently, we found that hypoxia-induced upregulation of *ANKZF1* was abolished upon silencing HIF-1α both by RT-qPCR and Western blot analyses (Fig. 5C and 5D)*. ANKZF1,* encoding ankyrin repeat and zinc finger domain-containing protein 1, was reported to play a role in the cellular response to hydrogen peroxide and in the maintenance of mitochondrial integrity under cellular stress conditions (van Haaften-Visser et al., 2017). To evaluate whether HIF-1α is capable of binding to the predicted four sites of the *ANKZF1* promoter, we performed chromatin immunoprecipitation (ChIP)-qPCR with an anti-HIF-1α antibody. Interestingly, we observed specific binding between HIF-1α and the *ANKZF1* promoter in *HIF-1α*^+/+^ hMSCs relative to *HIF-1α*^−/−^ cells (Fig. 5E). Subsequently, to query whether *ANKZF1* is directly activated by HIF-1α in hMSCs, we cloned the *ANKZF1* promoter region containing the four putative HIF-1α binding motifs upstream of the luciferase reporter, and found that the promoter of *ANKZF1* was indeed transcriptionally activated by hypoxia-induced HIF-1α (Fig. 5F). By contrast, we observed diminished *ANKZF1* promoter activity upon mutations of two core base pairs within each predicted binding sites of the *ANKZF1*, in particular within the site 4, as reflected by a massive reduction of luciferase activity (Fig. 5F). Overall, those data support a role for HIF-1α in positively regulating *ANKZF1* transcription in hMSCs.

Finally, we investigated whether ANKZF1 mediates the angiogenic functions of HIF-1α in hMSCs by silencing *ANKZF1* via CRISPR/Cas9-mediated knockout system (Fig. 5G). A decrease in protein level of ANKZF1 was confirmed by Western bloting of hMSCs transduced with *ANKZF1*-targeting sgRNA (Fig. 5H). Indeed, we observed a diminished capacity in cellular migration and in tube formation in *ANKZF1-*knockout hMSCs (Fig. 5I and 5J), which resembled the phenotypes we had observed in HIF-1α-depleted hMSCs (Fig. 2L and 2M). Collectively, our data suggested that *ANKZF1* is a novel target gene of HIF-1α that at least partially contributes to angiogenic modulation in hMSCs.

## Discussion

Ischemic conditions that reduce the supply of oxygen and nutrients can lead to severe injury, requiring vascular repair and blood flow recovery processes. However, the molecular programs intrinsic to human vascular cells that partake in ischemic vascular remodeling remain largely unknown. Herein, by using CRISPR/Cas9-mediated genome editing in human ESCs and directed differentiation, we generated HIF-1α-deficient human vascular cells to explore the effects of HIF-1α deficiency on neovascularization. We found that elevated expression of HIF-1α under hypoxic condition augments the angiogenic capability of human vascular cells *in vitro* and boosts the blood flow recovery at ischemic sites *in vivo*. We also unveiled that *ANKZF1*, by acting as a HIF-1α target gene in hMSCs, mediates the pro-angiogenic effect of HIF-1α. In sum, this study adds a layer to our understanding of the role of HIF-1α in human vascular cell homeostasis and angiogenesis, and identifies a new and potentially targetable mechanism for development of therapeutic interventions against ischemic diseases (Fig. 5K).

Ischemic diseases are often caused by blocked blood flow and associated with excessively high morbidity and mortality (Lei et al., 2021; Golledge, 2022). In the past, accumulating studies that used drugs or angiogenic factors to induce neovascularization generated disparate outcomes (Amsden, 2011). However, the majority were found to have limited effectiveness and undesirable side effects (Annex and Cooke, 2021). Therefore, efforts towards molecular profiling in human vascular cell models and in-depth mechanistic analysis aimed at decoding angiogenesis in human tissues are of both scientific and clinical importance. In the present study, we combined CRISPR/Cas9-mediated gene editing technology in hESCs cells with directed differentiation to establish human vascular cell models with genetic manipulation of HIF-1α. By generating this valuable experimental platform, we were able to functionally investigate causal mechanism underlying human vascular cell homeostasis and vascular regeneration, laying the groundwork for development of therapeutic treatments against ischemic diseases.

HIF-1α is generally considered to respond to oxygen level alterations and facilitate adaptation to hypoxia, oxidative stress, and metabolic changes by activating downstream genes (Zheng et al., 2022). However, the involvement of HIF-1α and its downstream molecular mechanism in different types of human vascular cells have not been fully explored. Here, by combining human pluripotent stem cell-derived human vascular cell and hindlimb ischemia mouse models, we uncovered how vascular regeneration and repair, normally supported by various human vascular cells, were abolished by HIF-1α deficiency *in vitro* and *in vivo*. Notably, with the exception of delayed restoration of blood flow, the persistent inflammatory responses reflected by enhanced numbers of CD45^+^ immune cells and elevated cytokine expression (e.g., TNF-α) in the ischemic zone were also observed in the *HIF-1α*^−/−^ implanted groups. In support of our findings, previous studies demonstrated that HIF-1α overexpression enhances immunomodulation ability by impairing dendritic cell differentiation, inducing suppressor macrophages, and enhancing resistance to NK cell-mediated lysis (Martinez et al., 2017; Cowman and Koh, 2022). Here, we revealed a crucial role of HIF-1α in directing angiogenic capacity of transplanted human vascular cells, thereby modulating the immune microenvironment *in vivo*, further demonstrating a potential causality between ischemia and inflammation, and supporting a potential therapeutic countermeasure against human ischemic diseases.

Numerous studies of blood vessel have mainly focused on endothelial cells and smooth muscle cells; however, the functions and mechanisms of vascular adventitia have remained understudied. In more recent work, MSCs were reported to reside within the tunica adventitial niche and to instruct vascular morphogenesis, repair, and self-renewal of vascular wall cells, processes that contribute to the local neovascularization in disease processes (Worsdorfer et al., 2017; Klein, 2020; Wang et al., 2022c). Here, based on RNA-seq data, we identified hMSC as the most sensitive cell type to hypoxia and HIF-1α deficiency compared to hVEC and hVSMC. Importantly, our ChIP-qPCR and luciferase reporter analysis support that *ANKZF1* is a novel HIF-1α target gene. ANKZF1, a cofactor binding to p97 (Stapf et al., 2011), was found to play a pivotal role in cellular response to hydrogen peroxide and in the maintenance of mitochondrial integrity under conditions of cellular stress (van Haaften-Visser et al., 2017). Here, we discovered that knockdown of *ANKZF1* in hMSCs mimicked the impaired angiogenetic phenotypes of *HIF-1α*^−/−^ hMSC under hypoxia. In support of our observation, a previous study showed that ANKZF1 plays an important role in angiogenesis in colon cancer (Zhou et al., 2019a). Collectively, our findings suggest that ANKZF1 serves as a downstream effector of HIF-1α and contributes to neovascularization in hMSCs.

In summary, we here, for the first time, generated HIF-1α-deficient models of the three major human vascular cells. Through the application of this valuable platform, we unraveled how HIF-1α-associated transcriptional programs boost angiogenesis, and identified *ANKZF1* as a novel HIF-1α target gene in human vascular cells. The new pathways and potential targets discovered in this study may facilitate development of new therapeutic approaches for ischemic diseases.

## Materials and methods

### Animal experiments

#### Teratoma assay

Teratoma assays were performed as previously described (Hu et al., 2020). In brief, ~5 × 10^6^ hESCs were injected into the groin cavities of NOD/SCID mice (male, 8 weeks old). After ~2 months, the teratomas were collected and analyzed by immunofluorescence staining with indicated markers.

#### Mouse hindlimb ischemia model induction and cell transplantation

BALB/c nude mice (8–10 weeks old) were used for hindlimb ischemia model construction as previous described (Yan et al., 2019). Briefly, mouse was anesthetized with isoflurane delivered at 2%. The proximal and distal femoral artery of the right hindlimb was ligated using 7-0 nonabsorbable suture. After surgery, 3 × 10^6^ hVECs + hVSMCs (3:1) or hMSCs were injected into the ischemic hindlimb in a 100 μL PBS and injected at six different locations immediately. For the control group, 100 μL of PBS without cells was injected. Blood perfusion was monitored every four days by the laser doppler blood perfusion (Moor instruments). Sixteen days after the ligation, hindlimb muscles were harvested for section staining.

### Cell culture

Human ESCs are cultured on mitomycin C-inactivated mouse embryonic fibroblast (MEF) feeder cells in CDF12 medium (DMEM/F12 medium containing 20% KOSR, 2 mmol/L GlutaMAX, 0.1 mmol/L NEAA, 1% penicillin/streptomycin, 55 μmol/L β-mercaptoethanol and 10 ng/mL FGF2), or on Matrigel in mTeSR medium (STEMCELL Technologies). hMSCs are cultured on 0.1% gelatin-coated plates in hMSC culture medium (MEMα medium supplemented with 10% FBS, 0.1 mmol/L NEAA, 1% penicillin/streptomycin, and 1 ng/mL FGF2). hVSMCs are cultured on 0.1% gelatin-coated plates in VSMC culture medium (50% DMEM/F12, 50% neurobasal, 2% B27, 1% N2, 1% penicillin/streptomycin, 55 μmol/L β-mercaptoethanol, 10 ng/mL PDGF). hVECs are cultured on collagen coated plates in EGM-2 medium (Lonza) supplemented with 10 nmol/L SB431542, 50 ng/mL VEGF, and 20 ng/mL FGF2. All cells were cultured in 37°C with 5% CO_2_. To induce stable expression of HIF-1α, cells were cultured in a hypoxia incubator containing 3% O_2_.

### Generation of *HIF-1α*^−/−^ hESCs

*HIF-1α* ^−/−^ hESCs were generated by CRISPR/Cas9-mediated gene knockout as previously reported with some modifications (Hu et al., 2020). Briefly, guide RNA targeting exon 2 of *HIF-1α* was cloned into gRNA-mCherry vector (HIF-1α-gRNA-mCherry) and electroporated into wild-type hESCs with pCAG-1BPNLS-Cas9-1BPNLS-2AGFP (Addgene, #87109) by 4D-Nucleofector (Lonza). After electroporation, cells were seeded on Matrigel-coated plates and treated with ROCK inhibitor (Tocris) in mTeSR. After 48 h of expansion, dual-positive cells were collected by FACS (BD FACS Aria II) and plated on MEF feeder cells in hESC medium. Emerging clones were manually picked into 24-well plates and then genomic DNAs of the clones were extracted for PCR and sequencing. Guide RNA sequences for gene editing and primers for clone identification are listed in Table S3.

### Generation of hVECs via directed differentiation from hESCs

hESCs were picked on Matrigel-coated plates and cultured in mTeSR medium. For directed differentiation to hVECs, hESCs were cultured in M1 medium containing IWP2 (3 mmol/L), BMP4 (25 ng/mL), CHIR99021 (3 mmol/L), and FGF2 (4 ng/mL), for 3 days. On the fourth day, M2 medium containing VEGF (50 ng/mL), FGF2 (20 ng/mL) and IL-6 (10 ng/mL) was used for another 3 days. The differentiated cells were harvested using Accumax and purified with hVEC specific markers (CD201 and CD144) by FACS. Dual-positive cells were collected as hVECs for future experiments. The antibody information was listed in Table S2.

### Generation of hVSMCs via directed differentiation from hESCs

hESCs were picked on Matrigel-coated plates and cultured in mTeSR medium for 4–5 days. The hESC clone with high quality was dissociated into single cells using TrypLE and seeded on Matrigel-coated plates with a concentration of 3 × 10^4^ cells/cm^2^. On the next day, culture medium was switched to M1 (VSMC basal medium with 25 ng/mL BMP4 and 8 μmol/L CHIR99021). On day 3, medium was switched to M2 (VSMC basal medium with 2 ng/mL Activin A and 10 ng/mL PDGF). On day 5, the cells were purified with CD140b antibody by FACS and cultured in VSMC basal medium with 10 ng/mL PDGF for future experiments. The antibody information was listed in Table S2.

### Generation of hMSCs via directed differentiation from hESCs

hESCs cultured on MEF feeders were digested and re-cultured in a low adhesion plate to obtain embryoid bodies. The embryoid bodies were transferred to Matrigel-coated plates and cultured in hMSC differentiation medium (MEMα medium supplemented with 10% Fetal Bovine Serum (FBS), 0.1 mmol/L NEAA, 10 ng/mL FGF2, 5 ng/mL TGF-β, and 1% penicillin/streptomycin). When cell density reaching 100% confluence, the fibroblast-like cells were passaged to gelatin-coated plated and maintained in hMSC culture medium (MEMα medium supplemented with 10% FBS, 0.1 mmol/L NEAA, 1 ng/mL FGF2, and 1% penicillin/streptomycin). Differentiated cells were purified by FACS of hMSC specific markers (CD105, CD90, and CD73) (Cheng et al., 2019; Liang et al., 2021). Triple-positive cells were collected as hMSCs for future experiments. The antibody information was listed in Table S2.

### Lentiviral CRISPR/Cas9-mediated knockout of *ANKZF1*

The CRISPR/Cas9-mediated gene knockout was performed as previously described (Zhang et al., 2022b). Briefly, the sgRNA targeting *ANKZF1* and nontargeting control (NTC) were cloned into lenti-CRISPRv2 vector (Addgene, #52961) containing an hSpCas9 expression cassette. For lentivirus production, HEK293T cells were co-transfected with lentiviral sgRNA plasmids along with lentiviral packaging vectors including psPAX2 (Addgene, #12260) and pMD2G (Addgene, #12259). Lentiviruses carrying sg-*ANKZF1* or control sgRNA were transduced into *HIF-1α*^+/+^ hMSCs. 48 h later, the cells were treated with puromycin (0.5 μg/mL) for around 5 days. The selected cells were collected for the subsequent analysis. The primer information was listed in Table S3.

### RNA extraction and analyses

Total RNA was extracted using TRIzol Reagent. One microgram of total RNA was reverse-transcribed to cDNA by using the GoScript Reverse Transcription System and oligo (dT) primer. PCR was carried out using Taq DNA Polymerase to detect the expression of pluripotency markers *OCT4*, *SOX2,* and *NANOG* in *HIF-1α*^+/+^ and *HIF-1α*^−/−^ hESCs. Human *GAPDH* was used as an internal control. qPCR was performed using a CFX384 Real-Time PCR system with iTaq Universal SYBR Green Super mix to verify the transcript changes of predict HIF-1α target genes. Human β*-actin* was used as an internal control. Primers used in this study are listed in Table S3.

### Immunofluorescence staining

Samples of cells seeded on coverslip or OCT embedding tissue sections were fixed in 4% paraformaldehyde, permeabilized in 0.4% Triton X-100 and blocked in 5% BSA-PBS. Primary antibodies were diluted in blocking buffer (5% BSA-PBS) and an incubation was conducted overnight at 4°C. After removal of the extra primary antibodies by PBS washing, samples were incubated with the corresponding fluorescence-labeled secondary antibodies at room temperature for 1 h. Nuclear DNA was labeled by Hoechst 33342. The fluorescent-positive cells or tissues were captured by laser scanning confocal microscopy and quantified using Image J software. The antibody information was listed in Table S2.

### Western blot

To detect the protein levels of HIF-1α and ANKZF1, cells were harvested in 2% SDS (*w*/*v*) solution supplemented with protease inhibitor cocktail (Roche) and boiled for 10 min. Protein concentration was measured by a BCA protein assay kit (Bicinchoninic acid). Twenty microgram total protein was loaded into SDS-PAGE gels for protein separation and then electro-transferred to PVDF membranes (Millipore). Following blocking with 5% (*w*/*v*) non-fat powdered milk (BBI Life Sciences) for 1 h at room temperature, the membrane was incubated with the corresponding primary antibodies overnight at 4°C. Then, the membrane was washed by TBST and incubated by HRP-conjugated respective secondary antibodies at room temperature for 1 h. Finally, image was generated by Image Lab 3.0 software (Bio-Rad) and analyzed with relative gray value by image J. The antibody information was listed in Table S2.

### ChIP-qPCR

ChIP-qPCR was performed according to previous protocols with slight modifications (Hu et al., 2020). Briefly, 1 × 10^6^ hMSCs pretreated with 3% O_2_ for 48 h were crosslinked by 1% (*v*/*v*) formaldehyde diluted in PBS for 13 min. The reaction was stopped by an incubation in 0.125 mol/L Glycine for 5 min at room temperature. After washes with PBS, cells were resuspended in ice-cold lysis buffer (50 mmol/L Tris-HCl, 10 mmol/L EDTA, 1% SDS, pH 8.0) for 5 min. After sonication by a Bioruptor® Plus device (Diagenode), supernatants were incubated overnight at 4°C with Protein A/G dynabeads (Thermo Fisher Scientific, 10004D) conjugated with anti-HIF-1α, or normal rabbit IgG. Subsequently, elution and reverse cross-linking were performed at 68°C for 3 h on a thermomixer. DNA was then isolated by the phenol–chloroform–isoamylalcohol extraction and ethanol precipitation method, and the purified DNA was used for qPCR detection. Primers used in this study are listed in Table S3.

### Plasmid construction and luciferase reporter assay

The promoter region (2,000 bp upstream of the transcription start site of *ANKZF1*) was obtained via PCR amplification and then cloned into PGL3-basic vector. The plasmids carrying the mutations of the binding sites within the promoter of *ANKZF1* were constructed using a Fast MultiSite Mutagenesis System (Transgen, Cat. No# FM201) and the mutagenic primers according to the manufacturer’s instructions. For single binding site mutation (Mut1, Mut2, Mut3, and Mut4), the corresponding primer pair was used; for multiple binding site mutation (Mut1-4), four pairs of primer were used together for amplification. The mutations were confirmed by DNA sequencing. Primers used in this study are listed in Table S3.

For luciferase reporter assay, hMSCs were cultured in 24-well plates and co-transfected with 1.0 μg plasmid of luciferase driven by *ANKZF1* promoter and 0.2 μg plasmid carrying Renilla using Lipofectamine® 3000 (Invitrogen). Forty-eight hours after transfection, cells were collected and relative luciferase activity was measured using Dual-Luciferase Reporter Assay System (T002, Vigorous Biotechnology Beijing Co., Ltd.).

### Transwell migration assay

For the transwell migration assay, 2 × 10^4^ cells were seeded on the top of 0.8 μm filters (Costar) in basal medium. Then, filters were placed into 24 culture plate wells containing complete medium. After 24 h of culture for hMSCs and hVECs or 48 h for hVSMCs, the filter inserts were fixed with 4% paraformaldehyde and then were stained by crystal violet for 30 min at room temperature. After washing, the migrated cells were photographed by light microscope and counted with Image J.

### *In vitro* tube formation assay

For the tube formation assay, 6 × 10^4^ cells were suspended in 600 μL complete medium and then seeded on Matrigel-coated 24-well plate. After 8–12 h, lattice-like vessel structures formed and the cells were then incubated with Calcein-AM (HY-D0041, Med Chem Express LLC) and examined by using fluorescence microscope.

### Masson’s trichrome staining

Ischemic hindlimb sections were washed three times with PBS, and then stained according to the protocol of Masson’s Trichrome stain kit (G1340, Solarbio). All images were captured using a digital pathology slide scanner (Aperio CS2, Leica). Infarcted scar size was calculated by using image J.

### CNV analysis

The genomic DNA was isolated from 1 × 10^6^*HIF-1α*^+/+^ or *HIF-1α*^−/−^ hESCs by using a DNeasy Blood & Tissue Kit (Qiagen). Quality control and sequencing were performed following standard protocols from Novogene Bioinformatics Technology Co. Ltd. Genome-wide CNV analysis was conducted as previously described (Yan et al., 2019). Raw reads were trimmed by the Trim Galore software (version 0.5.0) and clean reads were aligned to the UCSC hg19 human genome using bowtie2 software (version 2.2.9) (Langmead and Salzberg, 2012). R package HMMcopy (version 1.28.1) was implemented to calculate CNVs in each 0.5 Mb bin size (Ha et al., 2012).

### RNA-seq library construction and sequencing

Using the NEBNext® Poly (A) mRNA Magnetic Isolation Module, mRNA was isolated for RNA-seq. We constructed sequencing libraries using the NEBNext® Ultra™ RNA Library Prep Kit for Illumina following the manufacturer’s protocol. The libraries were sequenced on Illumina HiSeq X-Ten platforms with paired-end 150-bp sequencing. Quality control and RNA sequencing were done by Novogene Bioinformatics Technology.

### RNA-seq data processing

Raw data were trimmed by Trim Galore software (version 0.5.0). Clean data were mapped to the human reference genome (hg19) by HISAT2 software (version 2.0.4) (Kim et al., 2015). The reads mapped to gene were calculated using HTSeq software (version 0.11.0) (Anders et al., 2015). DEGs were calculated using the DEseq2 (version 1.30.1) (Love et al., 2014) with the cutoff of adjust *P* value less than 0.05 and |log_2_ (fold change)| more than 0.5. The FPKM (Fragments Per Kilobase of exon model per Million mapped fragments) of the gene was calculated using StringTie software (Pertea et al., 2015). GO terms and pathways enrichment analysis were performed by Metascape (Zhou et al., 2019b). The motif of HIF-1α was drawn using data from the JASPAR database (Castro-Mondragon et al., 2022). The predicted binding sites of HIF-1α on promoter of target genes were screened using MEME’s “motif scanning” function (Bailey and Elkan, 1994). 3 kb upstream of transcription start site was selected as promoter region. The DEGs are listed in Table S1.

## Statistical analysis

Data are shown as the mean ± SEM. Two-tailed Student’s *t* test was used for comparing the difference between groups. Multiple group comparisons were performed by one-way ANOVA followed by Tukey’s test or two-way ANOVA followed by Sidak’s test. GraphPad Prism 8.0 was used for statistical analysis. *P* < 0.05 is considered statistically significant.

## Supplementary Material

pwad027_suppl_Supplementary_Materials

pwad027_suppl_Supplementary_Table_S1

pwad027_suppl_Supplementary_Table_S2

pwad027_suppl_Supplementary_Table_S3

## Data Availability

Whole-genome sequencing and RNA-seq data have been deposited in the Genome Sequence Archive in the National Genomics Data Center, Beijing Institute of Genomics (China National Center for Bioinformation) of the Chinese Academy of Sciences, with accession number HRA003007. Other data or materials generated in this study are available from the corresponding authors upon reasonable request.

## Abbreviations

ChIP: Chromatin Immunoprecipitation
CNV: copy number variation
DEGs: differentially expressed genes
FACS: fluorescent-activated cell sorting
GO: Gene Ontology
hESCs: human embryonic stem cells
HIF-1α: hypoxia-inducible factor 1α
MSCs: mesenchymal stem cells
NI: non-ischemia
Isch: ischemia
PCA: principal component analysis
PCR: polymerase chain reaction
TNF-α: tumor necrosis factor-α
VECs: vascular endothelial cells
VSMCs: vascular smooth muscle cells
WT: wild-type

## Code availability

All the custom codes are available from the corresponding authors upon reasonable request.
