# Supplementary material for: Human ESC-derived vascular cells promote vascular regeneration in a HIF-1α dependent manner
Source: Protein Cell. 2023 May 9;15(1):36–51. doi: 10.1093/procel/pwad027 (PMC10762672; doi:10.1093/procel/pwad027)

Supplemental Materials

Figure S1. Cell type-specific transcriptional signatures in *HIF-1α*$^{+/+}$ and *HIF-1α*$^{-/-}$ human vascular cells.

(A) PCA analysis demonstrating the reproducibility of transcriptome expression profiles between replicates of hVECs, hVSMCs and hMSCs respectively.

(B) Box plots showing identification of cell type-specific genes in *HIF-1α*$^{+/+}$ hVECs, hVSMCs and hMSCs under normoxia.

(C) GO terms and pathways enrichment analysis of cell type-specific genes in *HIF-1α*$^{+/+}$ hVECs, hVSMCs and hMSCs under normoxia.

(D) Heatmap showing GO terms and pathways of upregulated genes in Hypoxia *HIF-1α*$^{+/+}$ vs. Normoxia *HIF-1α*$^{+/+}$ condition.

(E) Volcano plots showing DEGs between *HIF-1α*$^{+/+}$ cells under normoxic and hypoxic conditions, and between *HIF-1α*$^{+/+}$ cells and *HIF-1α*$^{-/-}$ cells under hypoxic condition. The numbers of upregulated and downregulated DEGs are marked in the upper right corner.

(F) GO terms and pathways enrichment analysis of HHRGs in hVECs, hVSMCs and hMSCs.

Supplemental Table legends

Table S1. DEGs identified by RNA-seq analysis of human vascular cells.

Table S2. Antibodies used in this study.

Table S3. Primers used in this study.

Supplemental figure 1

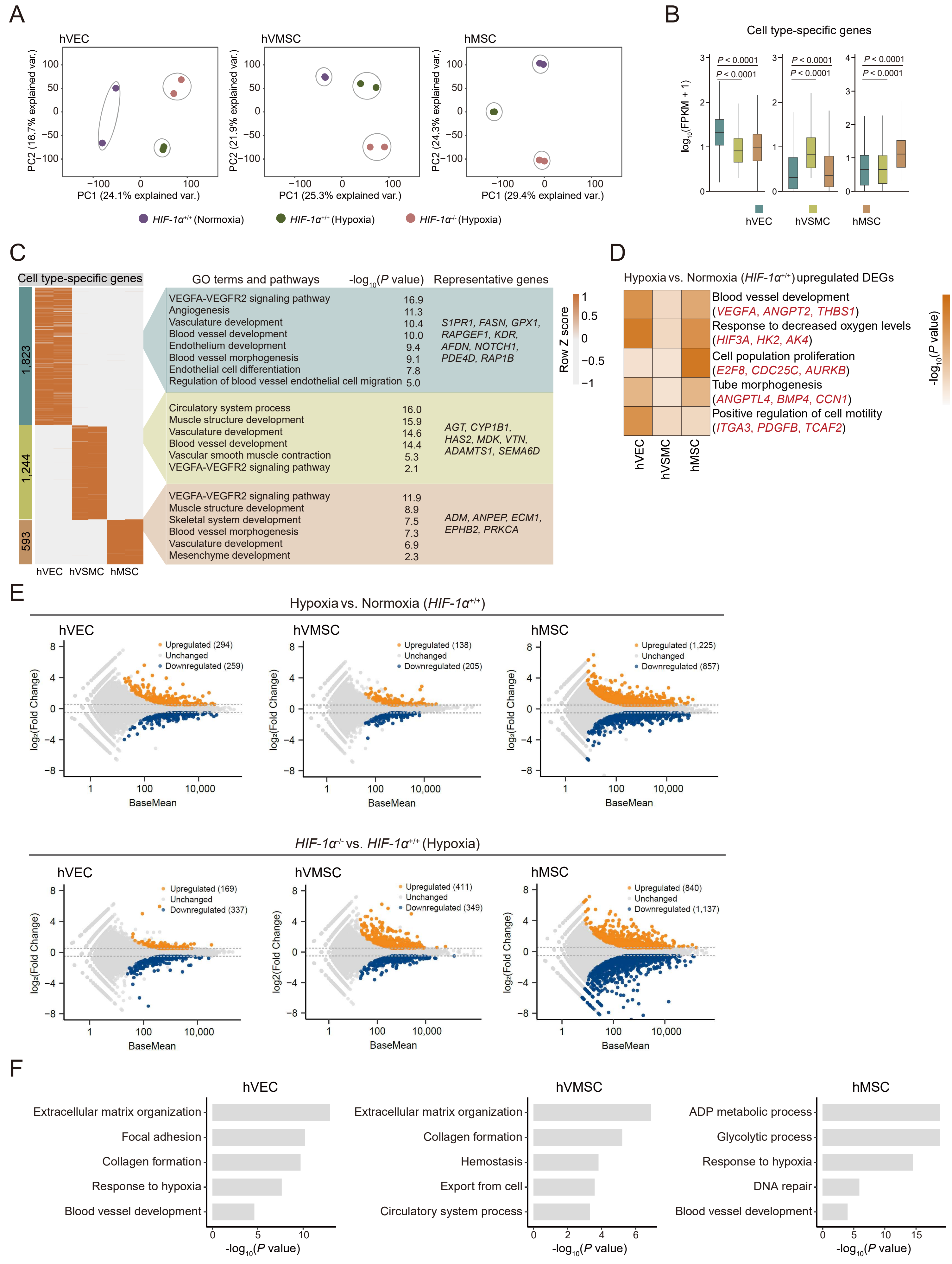

Supplement: pwad027_suppl_Supplementary_Materials [file pwad027_suppl_supplementary_materials.pdf]
